# Optimally biosynthesized, PEGylated gold nanoparticles functionalized with quercetin and camptothecin enhance potential anti-inflammatory, anti-cancer and anti-angiogenic activities

**DOI:** 10.1186/s12951-021-00836-1

**Published:** 2021-03-25

**Authors:** Priyadarshani S. Sadalage, Reshma V. Patil, Darshana V. Havaldar, Shruti S. Gavade, Ana Cláudia Santos, Kiran D. Pawar

**Affiliations:** 1 School of Nanoscience and Biotechnology, Shivaji University, Kolhapur, Maharashtra India; 2 Department of Pharmaceutical Technology, Faculty of Pharmacy, University of Coimbra, Coimbra, Portugal; 3 REQUIMTE/LAQV, Group of Pharmaceutical Technology, Faculty of Pharmacy, University of Coimbra, Coimbra, Portugal

**Keywords:** Gold nanoparticles, Functionalization of capped Gold nanoparticles, Anti-inflammatory activity, Cytotoxicity, Anti-angiogenesis

## Abstract

**Background:**

The development of nano delivery systems is rapidly emerging area of nanotechnology applications where nanomaterials (NMs) are employed to deliver therapeutic agents to specific site in a controlled manner. To accomplish this, green synthesis of NMs is widely explored as an eco-friendly method for the development of smart drug delivery system. In the recent times, use of green synthesized NMs, especially metallic NMs have fascinated the scientific community as they are excellent carriers for drugs. This work demonstrates optimized green, biogenic synthesis of gold nanoparticles (AuNPs) for functionalization with quercetin (QT) and camptothecin (CPT) to enhance potential anti-inflammatory, anti-cancer and anti-angiogenic activities of these drugs.

**Results:**

Gold nanoparticles were optimally synthesized in 8 min of reaction at 90 °C, pH 6, using 4 mM of HAuCl_4_ and 4:1 ratio of extract**:** HAuCl_4_. Among different capping agents tested, capping of AuNPs with polyethylene glycol 9000 (PG9) was found best suited prior to functionalization. PG9 capped AuNPs were optimally functionalized with QT in 1 h reaction at 70 °C, pH 7, using 1200 ppm of QT and 1:4 ratio of AuNPs-PG9:QT whereas, CPT was best functionalized at RT in 1 h, pH 12, AuNPs-PG9:CPT ratio of 1:1, and 0.5 mM of CPT. QT functionalized AuNPs showed good anti-cancer activity (IC_50_ 687.44 µg/mL) against MCF-7 cell line whereas test of anti-inflammatory activity also showed excellent activity (IC_50_ 287.177 mg/L). The CAM based assessment of anti-angiogenic activity of CPT functionalized AuNPs demonstrated the inhibition of blood vessel branching confirming the anti-angiogenic effect.

**Conclusions:**

Thus, present study demonstrates that optimally synthesized biogenic AuNPs are best suited for the functionalization with drugs such as QT and CPT. The functionalization of these drugs with biogenic AuNPs enhances the potential anti-inflammatory, anti-cancer and anti-angiogenic activities of these drugs, therefore can be used in biomedical application.
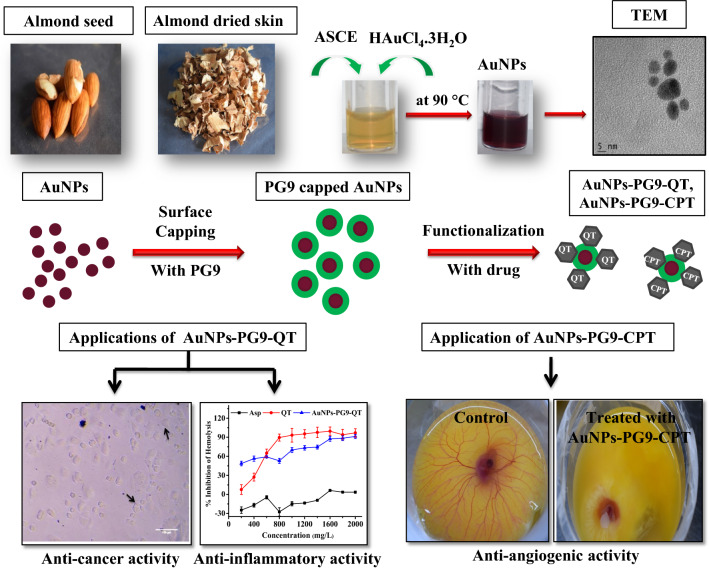

**Supplementary Information:**

The online version contains supplementary material available at 10.1186/s12951-021-00836-1.

## Background

Every year, tens of millions of people around the world are diagnosed with cancer, and it is estimated that more than half of them do not survive [1]. As estimated by GLOBOCAN (2018), the number of new cancer cases will reach 18.1 million, and cancer-related deaths will be close to 9.6 million [2]. As population aging continues in many countries, cancer will remain a major health problem around the globe [1]. Mainly, breast cancer is the second leading cause of death in women [3]. Most of the available cancer therapies respond with undesirable side effects on healthy cells [4]. Therefore, the prominent challenge in the cancer treatment is to develop a targeted drug delivery system using carrier tools from natural sources [5]. The size and shape of the drug vectors are of particular importance for the successful development of the drug delivery system [6]. Nanotechnology provides remarkable solutions to develop such tunable and suitable carrier platforms [7]. The development of nano delivery systems is a rapidly emerging area of nanotechnology application where nanomaterials (NMs) are employed to deliver therapeutic agents to a specific site in a controlled manner. In the recent times, the synthesis and use of NMs, especially metallic NMs have fascinated the scientific community as they are an excellent carriers for drugs [8], and can be modified in various ways for the controlled release of therapeutic payload into the cells for targeted delivery [9, 10]. It has been proven that conjugates of nano-size can penetrate deeper into tumors, are efficiently endocytosed, and work against primary and metastatic tumors [11]. Recently, gold nanoparticles (AuNPs) have received a great deal of attention as nano-carriers due to their unique anti-cancer [12] and anti-angiogenic [13] properties. Moreover, properties of AuNPs such as biocompatibility and ease of surface functionalization make them suitable for biomedical imaging [14] and as carrier in targeted drug delivery systems [15]. Also, the variety of organic ligands can be functionalized on the surface of AuNPs to enhance their properties [13].

For the development of nano-size drug delivery system, NMs synthesized by various methods such as chemical, physical and biological methods can be explored. In comparison, biological methods are preferred as they depend on minimum use of harmful chemical and generate the minimum toxic by-products [16]. In addition, biogenic synthesis methods offer control over the size and shape of NMs, facilitate mass production and reproducibility [17]. Nanomaterials such as AuNPs biosynthesized using plant [18], bacteria [19], fungi [12], yeast [20], algae [21] etc. are generally safer for use in biomedical applications, especially in cancer treatment, since they come from natural materials themselves. Among these, the plant-based methods provide new avenues for cost-effective, simple and rapid synthesis of metal NPs (MNPs) due to readily availability of plant materials. In addition, plant based active principles such as curcumin has also been employed for the synthesis of AuNPs [22, 23]. Since plant metabolites are economical, non-toxic [24] and accelerate the reduction of metal ions to MNPs, they show good potential to fulfill the high demand for MNPs for use in the biomedical applications [25]. Also they are suitable for surface functionalization due to their small size and stability [26].

The present study demonstrates plant mediated, optimized biosynthesis of AuNPs for functionalization with anti-cancer drugs namely quercetin (QT) and camptothecin (CPT). To accomplish this, almond seed coat extract (ASCE) was employed to optimize the biogenic synthesis. The optimization was done by studying the effects of variations in different synthesis parameters such as incubation time, temperature, pH, ratio of extract**:**precursor and precursor concentration. Next, the optimally synthesized AuNPs were characterized using various spectroscopic and imaging techniques, surface capped with polyethylene glycol 9000 (PG9) and then functionalized with QT and CPT. Further, anti-cancer and anti-inflammatory activities of QT functionalized AuNPs were evaluated against Human Breast Cancer Cell line MCF-7 (MCF-7) using 3-(4,5-dimethylthiahiazol-2-yl)-2,5-diphenyl tetrazolium (MTT) assay and by inhibition of hemolysis of red blood cell (RBC) membrane, respectively. In addition, chorioallantoic membrane (CAM) based assay for the assessment of in vivo anti-angiogenic activity was employed to evaluate the potential of CPT functionalized AuNPs to inhibit the formation of blood vessel in chick embryos.

## Results and discussion

### Optimization of biogenic synthesis of AuNPs

**Fig. 1:**
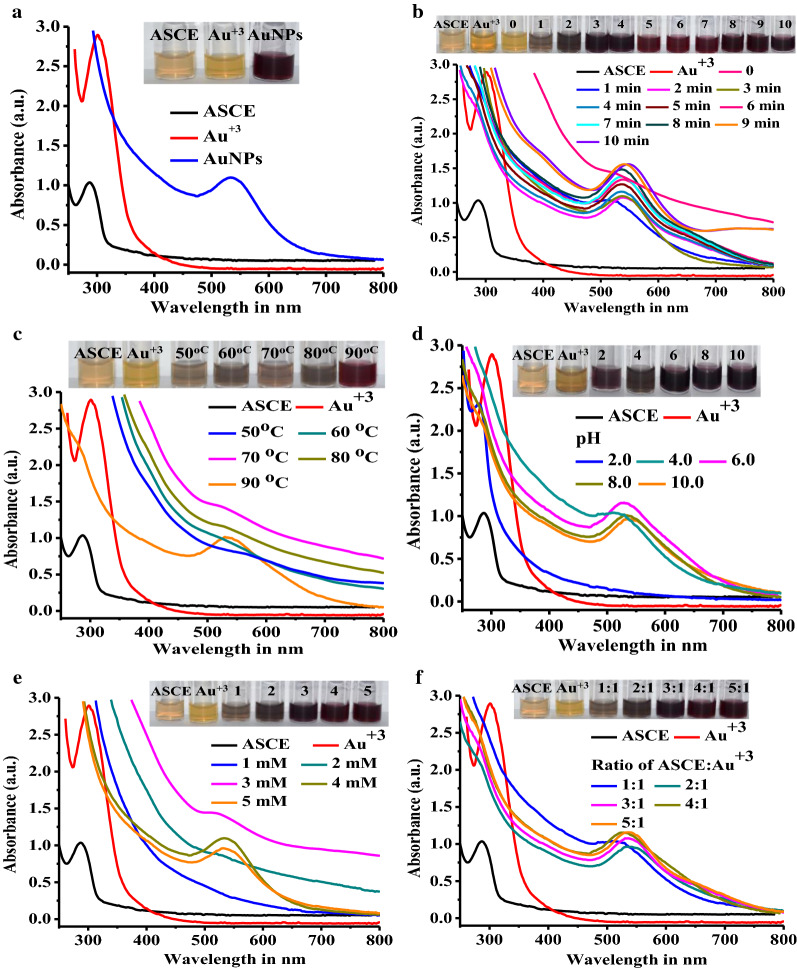
Optimization of almond seed coat extract mediated biogenic synthesis of AuNPs. **a** Color change of initial biogenic synthesis reaction, and UV–Vis absorption spectra showing the effects of **b** incubation time, **c** incubation temperature, **d** pH, **e** concentration of HAuCl_4_ and **f** ratio of extract:precursor on biogenic synthesis of AuNPs

Biogenic synthesis of MNPs is gaining importance for being simple, eco-friendly, lower toxicity and good biocompatibility of biogenic MNPs [27]. Since plants are easily available, safe to handle and consist of broad range of metabolites, they are preferred as sources of capping and reducing agents for biogenic synthesis [28]. In the present study, the initial reaction between ASCE and HAuCl_4_ precursor solution at 90 °C for 10 min turned the reaction color from pale yellow to light purple and then dark ruby red indicating the synthesis of AuNPs due to reduction of Au^+3^ to Au^o^ (Fig. 1a). The ASCE mediated AuNPs exhibited a strong SPR peak at 530 nm which is in agreement with earlier few studies that reported phytomediated synthesis of AuNPs from *Mangifera indica* leaf [29], *Magnolia kobus* leaf extracts [30] and *Solanum torvum* [31]. The literature survey indicates that the optimization of different reaction parameters is needed to control the size and shape of the biogenic NMs [32–34]. Therefore, we next optimized the ASCE mediated AuNPs synthesis by studying the effects of variation in different reaction parameters on synthesis.

### Effect of incubation time on AuNPs synthesis

Figure 1b shows the effect of incubation time on ASCE mediated AuNPs synthesis. At 0 min, the reaction was pale yellow with a broad, hyperbolic SPR peak that indicated the slow interaction of biomolecules with precursor solution. As the reaction proceeded from 0 to 1 min., color turned light purple due to ASCE mediated initiation of bio-reduction of Au^+3^ to AuNPs. Further continuation of reaction from 1 to 4 min gradually turned the reaction color from light purple to dark purple with broad SPR peak at 530 nm demonstrating AuNPs synthesis. Thereafter, the intensity of SPR peak at 530 nm and ruby red color gradually increased when incubation was extended from 5 to 8 min indicating the complete reduction (Fig. 1b). When incubation time was further increased to 9 and 10 min, SPR peaks at 530 nm were found shifted to 541 and 548 nm, respectively. Thus, the variation in reaction time clearly demonstrated that ASCE requires 1 min to initiate the reaction and completes in 8 min. Therefore, the ASCE mediated AuNPs synthesis demonstrated in this study was rapid when compared with earlier studies [27, 35].

### Effect of temperature on AuNPs synthesis

In addition to reaction time, the reaction temperature has also been proven as one of the important factors that influence the rate of nucleation and reaction, growth, yield, and other properties of AuNPs [33]. As the temperature increases, the rate of reaction accelerates due to increase in kinetic energy and nucleation resulting in the formation of small nuclei [30] whereas, low temperature requires more time for AuNPs synthesis [34]. In the view of this effect of reaction temperature, we varied the reaction temperature in the range of 50–90 °C for optimum AuNPs synthesis (Fig. 1c). On incubating the reactions in the range of 50–80 °C, no dark ruby red color with sharp SPR peaks characteristic of AuNPs were observed (Fig. 1c). This suggested that at 50–80 °C, there was incomplete or slow reduction of Au^+3^ ions to Au°. Noteworthy, when the reaction was incubated at 90 °C, it turned dark ruby red and showed typical SPR at 530 nm indicating the optimum reduction of Au^+3^ to smaller sized AuNPs (Fig. 1c). Our observation coincides with previous few studies that also reported the optimum biogenic syntheses of AuNPs at higher temperatures using extracts of various plants such as *Tiliacorat riandra* [36], *Magnolia kobus* [30] and *Chenopodium album* [37].

### Effect of pH on AuNPs synthesis

It is known that pH of the synthesis reaction also plays a substantial role in reduction, synthesis and stabilization of NPs. It can be explained by the fact that increase in the reaction pH promotes the capping of NP’s surface more efficiently and supports the formation of smaller NPs [38]. In the present study, when precursor solution was added to ASCE and pH of the reaction mixture was measured before embarking into reaction, it was found to be 6. Therefore, for the study of influence of pH, we studied the effect of variation of pH 6 ± 4 i.e. in the range of 2–10 (Fig. 1d). At high acidic pH 2, synthesis was not observed whereas, at pH 4, reaction turned slightly purple but no characteristic SPR peak of AuNPs was observed. This indicated that high acidic condition did not favor the synthesis of AuNPs as it could have inactivated the biomolecules of ASCE functioning as reducing and capping agents. This was quite in congruence with the observation made by Pimprikar and co-workers (2009). These authors demonstrated that the extreme acidic conditions (pH 2) of reaction inactivate the metabolites of extract and are likely to reduce the synthesis [20]. In our case, when the pH was increased to slight acidic condition of 6, the reaction changed from light yellow to dark ruby red with intense and narrow SPR peak at 529 nm which clearly demonstrated AuNPs synthesis (Fig. 1d). Though further increase in pH to 8 and 10 turned reactions dark ruby red, the SPR peaks were broadened and accompanied with red shifts at 540 and 545 nm respectively. Moreover, intensities of the SPR peaks were reduced demonstrating the synthesis of large sized AuNPs. The observed red shifts in reactions at alkaline pH could have occurred due to plasmon coupling between aggregated NPs [39]. Thus, we found that pH 6 was optimum for ASCE mediated synthesis of AuNPs.

### Effect of precursor concentration on AuNPs synthesis

In order to study how the variation in the precursor HAuCl_4_ concentration influences the AuNPs synthesis, it was varied in the range of 1–5 mM and analyzed (Fig. 1e). Precursor in the range of 1–3 mM turned reactions light yellow but AuNPs were not synthesized as no sharp and intense characteristic SPR peaks were observed. However, when reaction contained 4 mM HAuCl_4_, dark ruby red color was observed with sharp and intense SPR peak at 530 nm indicating complete reduction of Au^+3^ ions to AuNPs (Fig. 1e). In case of use of 5 mM of HAuCl_4_, the SPR peak was broadened and slightly red shifted to 536 nm with decreased intensity. These results are in agreement with the previous report that states that the concentration of biomolecules of plant extract may not be sufficient to reduce high concentration of HAuCl_4_ [20].

### Effect of extract to precursor ratio on AuNPs synthesis

In addition to incubation time, temperature, pH and concentration of precursor, the ratio of extract to precursor is also known to influence the synthesis of MNPs such as AuNPs [20]. In the light of this information, we varied the ASCE:precursor ratio in the range of 1:1–5:1 and analyzed its effect of AuNPs synthesis (Fig. 1f). At 1:1 ratio, the reaction turned light purple without distinct SPR peak at 530 nm. Later, when ratio was increased to 2:1 and 3:1, it developed a light and dark purple color with broad SPR peaks at 540 and 537 nm, respectively whereas, the SPR peak of reaction with 4:1 ratio was found proportionally more intense at 530 nm indicating the optimum synthesis (Fig. 1f). In case of 5:1 ratio, the intense SPR peak was shifted to 537 nm demonstrating the synthesis of large sized AuNPs. Our observation of increased NPs size due to increased concentration of extract is in agreement and goes well with earlier report. Khalil and colleagues found that the reaction with higher volumes of the extract shows slightly decreased intensity and red shift of SPR indicating the saturation in the reduction of Au^+3^ [38].

Thus, based on these studies of effects of variation in different synthesis parameters, it was found that AuNPs were optimally synthesized at 8 min of reaction, 90 °C, pH 6, 4 mM of HAuCl_4_ and 4:1 ratio of ASCE:HAuCl_4_.

### Characterization of AuNPs

**Fig. 2:**
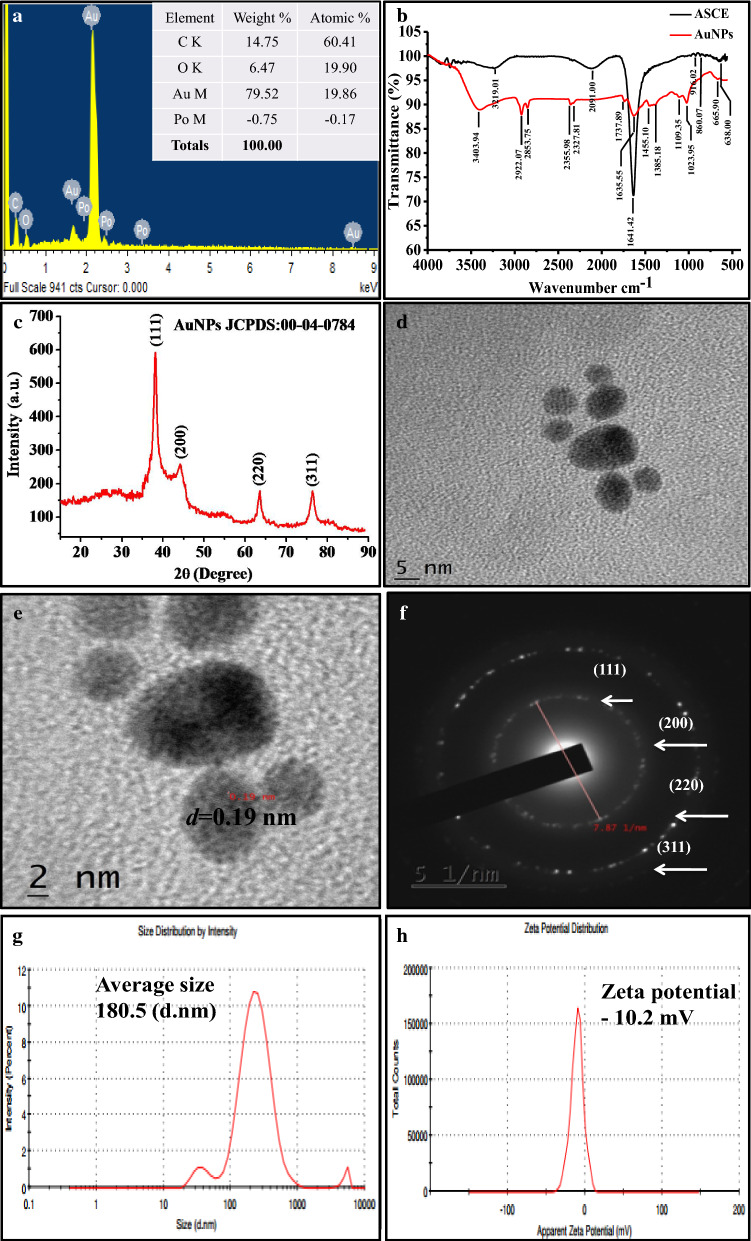
Characterization of biogenic AuNPs by **a** EDS, **b** FTIR, **c** XRD, **d** TEM, **e** HR-TEM, **f** SAED, **g** DLS and **h** Zeta potential pattern analyses

For the characterization of AuNPs using elemental analysis, the recording of EDS spectra illustrated the presence of elemental gold (Au) (Fig. 2a). The EDS profile showed a strong signal of Au followed by carbon (C), oxygen (O) and minor peak of polonium due to AuNPs surface bound organic biomolecules from ASCE [40]. Gold peak was observed approximately at 2.2 keV which is typical for the absorption of metallic Au nano-crystallites due to SPR. The EDS profile demonstrated that Au contributed by 79.52 weight % to AuNPs whereas other detected elements namely C and O were found to contribute by 14.75 and 6.47 weight %, respectively. The higher elemental contribution by Au clearly demonstrated that synthesized NPs were indeed AuNPs whereas, the presence of C and O related signal confirmed the biogenic nature of AuNPs.

The FTIR spectra of ASCE (Fig. 2b) showed strong band of IR stretchings at 3219.01 and 2091.00 cm^−1^ corresponding to OH groups bonded to the aromatic ring and C–C stretching, respectively (Additional file 1: Table S1) whereas, IR band at 1641.42 cm^−1^ in the spectrum of ASCE and at 1635.55 cm^−1^ in IR spectrum of AuNPs were assigned to N–H bending vibration of primary amines. The capping of ASCE biomolecules onto the AuNPs surface could have broken most of the H bonds between the N–H groups that could have led to observed marginal shift [27] and decrease in intensity of IR band [41]. The FTIR spectra of AuNPs showed the major characteristic peak at 3403.94 cm^−1^ related to O–H and N–H stretching in amine group while other peaks at 2922.07 and 2853.75 cm^−1^ were attributed to C–H stretching vibrations. The intense broad absorbance at 2355.98 and 1023.95 cm^−1^ corresponded to C=C and C–N stretching vibration of aliphatic amines or to alcohols/phenols, respectively. The emergence of new peak at 1737.89 cm^−1^ corresponding to stretching vibrations of carbonyl (C=O) indicated the conversion of alcoholic group into aldehyde to reduce Au^+3^ to Au^o^ [42]. The additional minor peaks of AuNPs corresponded to different bending and stretching vibrations of the bioactive molecules as shown in Additional file 1: Table S1. Thus, our FTIR based investigation indicated the involvement of amines, alcohols, phenols, and aromatic groups of ASCE as reducing and capping agents in synthesis of AuNPs.

The recording and analysis of XRD pattern (Fig. 2c) of AuNPs for the study of crystalline nature showed four intense peaks of 2θ values in the range from 10–90. These four distinct diffraction peaks at 36.58°, 42.71°, 61.90° and 74.78° corresponded to (111), (200), (220) and (311) crystallographic planes. The Bragg reflections closely matched to the reported reference values of Joint Committee on Power Diffraction Standards (JCPDS) card No. 04–0784 which demonstrated that AuNPs resembled the face-centered cubic (FCC) structure of metallic Au and composed of pure crystalline Au. The observed XRD pattern coincided with that previously reported by Rajakumar et al., (2016) who stated that AuNPs with FCC structure were oriented along the (111) plane[18].

Further, imaging the morphologies and size by TEM indicated the spherical and oval shape of AuNPs with an average size in the range of 5–15 nm (Fig. 2d). The HR-TEM analysis also demonstrated and confirmed the spherical morphology and internal polycrystalline nature of AuNPs with 0.19 nm of d-spacing in the lattice fringes (Fig. 2e). The TEM based SAED pattern (Fig. 2f) showed diffraction rings associated with the (111), (200), (220) and (311) atomic planes. Spots corresponding to various orientations of the nanocrystals which appeared inside the concentric rings clearly showed crystalline nature of AuNPs that matched with XRD pattern as shown in Fig. 2c. Thus, the HR-TEM and SAED analyses clearly confirmed the single crystal structure of AuNPs with FCC phase. The size of AuNPs and their distribution in solution analyzed by DLS (Fig. 2g) indicated the poly-dispersed nature of AuNPs with average hydrodynamic diameter of 180.5 nm (d.nm). The average zeta potential of -10.2 (Fig. 2h) mV showed good stability and negative charge on AuNPs surface due to capping by biomolecules of ASCE.

### Surface capping of AuNPs

In the present study, our initial attempts to functionalize neat AuNPs with QT and CPT failed as they were not efficiently functionalized. This could have occurred due to the lack of suitable surface groups or ligands for exchange with these drugs. Therefore, in order to facilitate efficient functionalization, we first surface capped AuNPs with polymers such as PG6, PG9 and PVP K25. Additional file 1: Figure S1 indicate the UV–Vis absorption spectra of surface capping reactions of AuNPs with PG6, PG9 and PVP K25. The reactions of AuNPs with tested capping agents resulted in the red shift of absorption bands in the range of 535–538 nm. The resultant slight decrease in color intensities indicated the modifications in the AuNPs surface due to surface capping (Additional file 1: Fig. S1). It was observed that AuNPs surfaced capped with PG9 displayed a characteristic peak at 535 nm with high absorbance as compared to AuNPs surfaced capped with PG6 and PVP K25 (Additional file 1: Fig. S1). These peaks related to the surface plasmon and increase in intensity was observed due to increased density of the reaction mixture by capping with polymers (Additional file 1: Fig. S1). Similar effect of capping agents has been reported by Oh and coworkers [43]. It was reported that red shift of SPR band in capping reaction is a consequence of increased refractive index surrounding the AuNPs due to the modification of PEG molecular chain length [44]. Moreover, the slight variation in absorption values of AuNPs capped with PG6 (Additional file 1: Fig. S1) and PG9 (Additional file 1: Fig. S1) indicated that AuNPs were coated with PEG of different molecular weight [45]. Considering these, it was concluded that PG9 was comparatively more suitable for surface capping of AuNPs and, therefore was selected for further optimization of functionalization with anti-cancer drugs.

### Optimization of functionalization of AuNPs-PG9 with QT and CPT

Though the initial reaction between PG9 capped AuNPs (AuNPs-PG9) and QT solution at 50 °C for 2 h changed the reaction from dark ruby red to light ruby red with strong SPR peak at 536 nm indicating functionalization (Additional file 1: Fig. S2a), it was worth investigating how the variation in different reaction conditions influences the functionalization. Additional file 1: Figure S2 represents the effect of incubation time on functionalization of QT with AuNPs-PG9. It was found that increase in the reaction time was accompanied by increase in SPR peak intensity. The SPR peak intensity of functionalization reaction was gradually increased with increased reaction time from 0 to 30 min (Additional file 1: Fig. S2b). Further increase from 45 to 60 min also resulted in increased absorbance with sharp, narrow SPR peak at 535 and 533 nm, respectively. After 60 min of reaction (75–120 min), the narrow SPR peak started broadening with reduced SPR peak intensities. Previously, it has been reported that inter-particle spacing decreases with increased reaction time that subsequently leads to slight red shift indicating surface functionalization [46]. Therefore, in our case, 60 min was found optimum reaction time for functionalization of QT with AuNPs-PG9.

The variation in reaction pH in range of 4–7 demonstrated that pH 4 and 5 result in red shift and broad SPR peak (539 nm) whereas; pH 6 and 7 result in gradual increase in SPR intensity with blue shift (Additional file 1: Fig. S2c). It has been stated that QT functionalized with NPs exhibit maximum stability at pH between 6 and 7. Within this pH range, the interaction between AuNPs-PG9 and QT occurs through hydrogen bond formation since both maybe deprotonated and protonated in alkaline and acidic condition, respectively; that may influence the H-bonding [47].

In addition to variation in incubation time and pH of the reaction, the effect of variation in QT concentration on functionalization was also studied by varying the AuNPs-PG9:QT ratio in the range of 1:1–1:4 and monitored by UV–Vis. spectroscopy (Additional file 1: Fig. S2d). When ratio was increased from 1:1 to 1:4, the SPR peak intensities linearly increased with slight red shifts from 530 to 535 nm. It is stated that the lower concentration of molecules to be functionalized does not effectively built-up around the NPs whereas, high enough concentration favors the functionalization of surface capped NPs [48]. Since highest SPR peak intensity with red shift was noted for reaction with ratio 1:4, it was consider optimum for preparation of AuNPs-PG9 functionalized with QT (AuNPs-PG9-QT).

The effect of variation of reaction temperature in range of 30–70 °C on AuNPs-PG9-QT synthesis resulted in comparatively less intense and broad SPR peaks at 30 and 50 °C (Additional file 1: Fig. S2e). This may be due to the increased size and hydrogen bond formation between AuNPs-PG9 and QT [49]. On the contrary, more intense and sharp SPR band was observed at 533 nm when reaction was incubated at 70 °C indicating it as the optimum temperature for AuNPs-PG9-QT synthesis.

Besides optimizing the AuNPs-PG9:QT ratio, the effect of variation in the concentration of QT in the range of 400–1600 ppm was also investigated in the present study (Additional file 1: Fig. S2f). We observed that SPR intensity was gradually increased when QT concentration was increased from 400 to 1200 ppm. At QT > 1200 ppm i.e. in the range of 1400–1600 ppm, the SPR bands turned broad with slight red shifts. The broadening and shifting of absorption band could have occurred due to the formation hydrogen bonds in AuNPs clusters [50]. The highly intense and sharp SPR band was recorded when reactions contained 1200 ppm of QT; therefore, we considered it as the best concentration for AuNPs-PG9-QT synthesis. Thus, based on variations in the different conditions of functionalization reaction, we found that incubation time of 60 min, pH 7, AuNPs-PG9:QT ratio of 1:4, temperature of 70 °C and 1200 ppm of QT were the most optimum conditions for functionalization of AuNPs-PG9 with QT.

In addition to functionalization with QT, AuNPs-PG9 were also tested for their potential to get functionalized with CPT. When the effect of concentration of CPT in the range of 0.1–0.5 mM was tested, 0.5 mM CPT resulted in higher SPR intensity and shift to 535 nm indicating the optimum functionalization (Additional file 1: Fig. S3a). Further, optimization of reaction time in the range of 1–6 h indicated 1 h as optimum time as SPR peak was found shifted to 536 nm. This SPR shift clearly demonstrated that 1 h reaction time was optimum for functionalization of CPT with AuNPs-PG9 (Additional file 1: Fig. S3b). Likewise, variation of pH of the reaction in the range of 2–12 showed that the optimum functionalization of CPT takes place in basic condition (pH 12), since the reaction showed the shift of SPR to 539 nm (Additional file 1: Fig. S3c).

Thus, based on variations in the different conditions, we found that incubation time of 1 h, pH 12, AuNPs-PG9:CPT ratio of 1:1, RT and 0.5 mM of CPT were the most optimum conditions for functionalization of AuNPs-PG9 with CPT.

### Characterization of AuNPs-PG9-QT and AuNPs-PG9-CPT

**Fig. 3:**
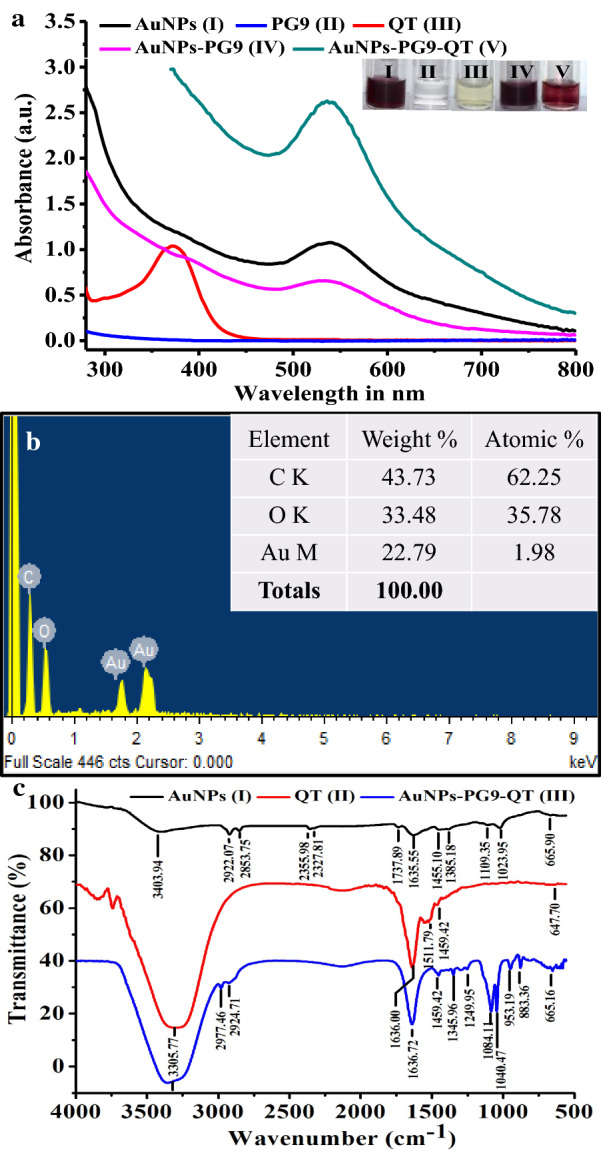
Characterization of polyethylene glycol 9000 capped and quercetin functionalized AuNPs by **a** UV–Vis, **b** EDS and **c** FTIR spectroscopy

The large scale synthesis of AuNPs-PG9-QT and its comparison with neat AuNPs and AuNPs-PG9 by UV–Vis spectroscopy showed typical SPR peaks at 535, 530 and 531 nm, respectively (Fig. 3a). The elemental analysis of AuNPs-PG9-QT by EDS (Fig. 3b) exhibited the typical signal of metallic Au nano-crystallites at 2.2 keV. When compared with EDS of neat AuNPs, the EDS spectrum of AuNPs-PG9-QT showed that elemental Au accounted for 22.79 weight %. Noteworthy, C and O contents were found increased from 14.75 and 6.47% in AuNPs (Fig. 2a) to 43.73 and 33.48% in AuNPs-PG9-QT (Fig. 3b), respectively. The increased contents of C and O complex suggested that considerable amount of Au atoms were capped, covered with large number of QT molecules demonstrating successful functionalization. The similar changes in EDS spectrum of functionalized NPs has been reported previously [47]. Further analysis of AuNPs-PG9-QT by FTIR confirmed the functionalization as several bands present in the IR spectra of AuNPs and QT (Fig. 3c) were shifted, however their characteristic signatures were maintained. The FTIR spectrum of AuNPs-PG9-QT (Fig. 3c) showed characteristic peaks at 3305.77 cm^−1^ corresponding to free OH bond vibration whereas, peaks at 2977.46 and 2924.71 cm^−1^ were assigned to the stretching vibration of C-H suggesting the inter-molecular hydrogen bonding between QT and PG9 capped AuNPs. In addition, other major and minor characteristic peaks were also detected in AuNPs-PG9-QT in the range of 1600–650 cm^−1^. The stretching vibrations of these peaks and their functional groups are presented in Additional file 1: Table S2. The absorption band representing aromatic group of QT (at 1511.79 cm^−1^) was completely disappeared in spectrum of AuNPs-PG9-QT. The IR band at 1249.95 cm^−1^ in FTIR spectrum of AuNPs-PG9-QT (Fig. 3c) could be attributed to C–O stretching vibrations suggesting the formation of ester bond for binding of carboxyl end of PG9 with the catechol group of QT [51]. Moreover, presence of C-O, C = O, C–H and N–H related stretching vibration confirmed the presence of bound PG9. The prominent IR peaks related to phenol and aromatic group represented the effective binding of QT to form AuNPs-PG9-QT conjugate (Fig. 3c and Additional file 1: Table S2) which is well in agreement with the existing data in the literature [52].

**Fig. 4:**
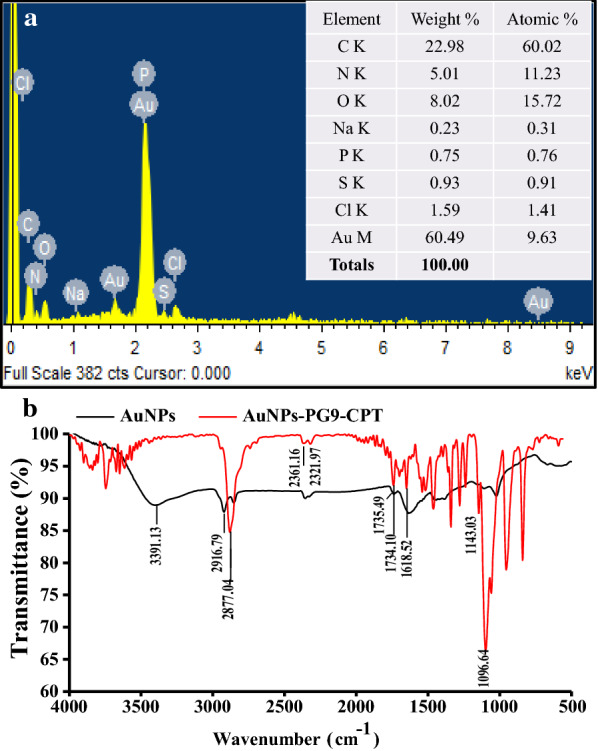
Characterization of polyethylene glycol 9000 capped and camptothecin functionalized AuNPs by **a** EDS and **b** FTIR spectroscopy

In case of characterization of AuNPs-PG9-CPT by EDS, highest peak corresponding to AuNPs was recorded at 2.2 keV while other peaks at 0.2, 0.4 and 1KeV corresponding to C, O and Na respectively were also recorded. Based on EDS analysis, Au was found to contribute by 60.49% to the weight of AuNPs-PG9-CPT (Fig. 4a). The FTIR spectrum of AuNPs-PG9-CPT showed different IR peaks at different positions for various functional groups. The peak at 1735 cm^−1^ corresponded to carbonyl stretching for cyclic lactone while the band at 1143 cm^−1^ corresponded to C–C (=O)–O stretching vibration. In addition, the IR band at 1618 cm^−1^ was ascribed to C=O stretching vibration whereas the peaks at 2852, 2361 and 1095 cm^−1^ were assigned to C=H stretching, N=C=O stretching and C=O stretching, respectively. Also, the band at 2916 cm^−1^ was found to correspond to C-H vibrational stretching of the methylene groups of the protein (Fig. 4b).

### Anti-cancer activity of AuNPs-PG9-QT

**Fig. 5:**
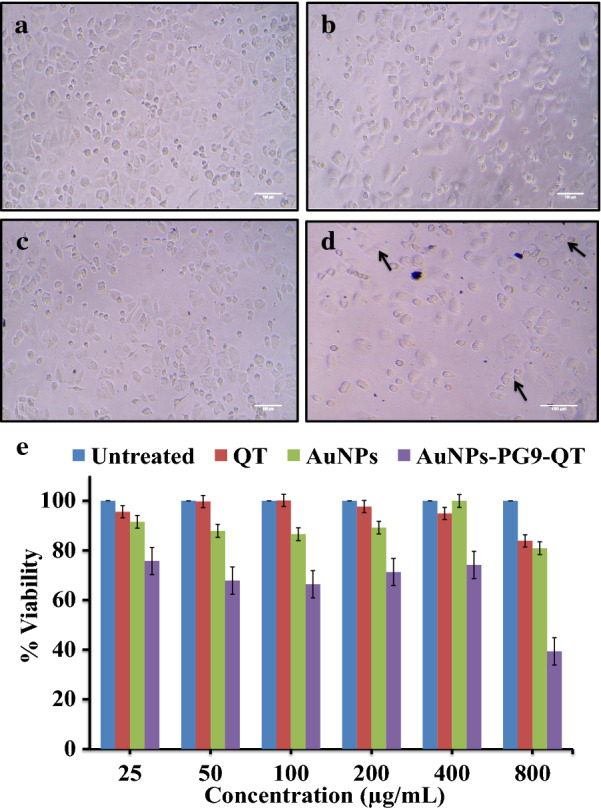
Anticancer effect of polyethylene glycol 9000 capped and quercetin functionalized AuNPs. Morphological changes to MCF-7 cell line after treatment with **a** control, **b** only quercetin, **c** neat AuNPs, **d** polyethylene glycol 9000 capped Quercetin functionalized AuNPs and **e** effect of different concentration of Quercetin, AuNPs, and polyethylene glycol 9000 capped and Quercetin functionalized AuNPs on cell viability of MCF-7 cell line. (In **d** arrows indicate that AuNPs-PG9-QT treated cells were ruptured and observed for irregular shape)

In order to assess how the functionalization of QT with AuNPs-PG9 influences the anti-cancer potential, the anti-cancer activities of AuNPs, QT and AuNPs-PG9-QT were studied by employing MTT assay on cancer cell line MCF-7 and compared. To this end, AuNPs-PG9-QT in the range of 20–800 μg/mL were treated with MCF-7 cells and compared with untreated (control) cells (Fig. 5e). In comparison to AuNPs and QT which showed marginal activity, AuNPs-PG9-QT in the tested range significantly reduced the cell viability indicating good anti-cancer activity with IC_50_ value of 687.44 µg/mL (Table 1). This observation demonstrated that the functionalization of QT with AuNPs-PG9 does not reduce or loses the activity; rather the activity of functionalized preparation (AuNPs-PG9-QT) was enhanced significantly. In line with our observation, recently Rameshthangam and Chitra had also observed that the anti-cancer activity of PEG capped nickel NPs against MCF-7 cell line enhances when functionalized with QT [52]. Similar enhanced anti-cancer activity for arginine encapsulated PEG-coated magnetic has also been reported [53]. In the present study, the anti-cancer activity of AuNPs-PG9-QT was also evident from the change in morphologyof MCF-7 cells (Fig. 5a–d). As compared to untreated cells, AuNPs-PG9-QT treated cells were ruptured, few changed their shape from spindle to circular or irregular, and the number of adhered cells was also decreased (Fig. 5d). Our observation on changed cell morphology due to treatment of QT functionalized NPs is in agreement with that of Firoozeh et al. who studied the effect of QT encapsulated in solid lipid NPs [54].

**Table 1:** Effect of PG9 capped and quercetin functionalized AuNPs on toxicity to MCF-7 cell line

Sample	MCF-7 cell line IC_50_ 24 h
Quercetin	NA (Low toxicity at highest concentration)
Biogenic AuNPs	NA (Low toxicity at highest concentration)
PG9 capped and Quercetin functionalized AuNPs	687.44 µg/mL

### Anti-inflammatory activity ofAuNPs-PG9-QT

**Fig. 6:**
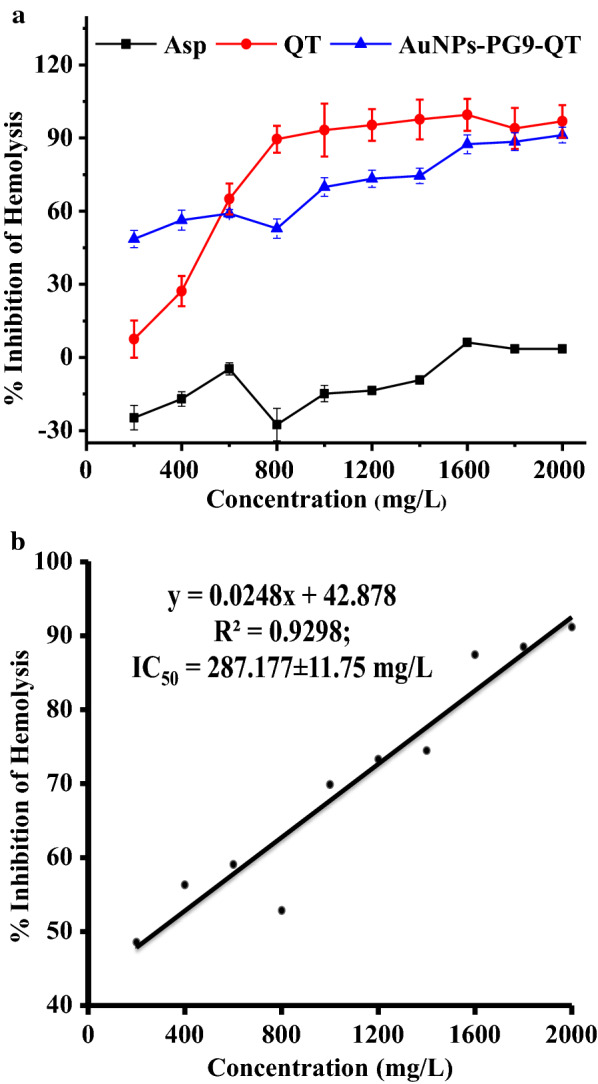
Anti-inflammatory activity of polyethylene glycol 9000 capped and Quercetin functionalized AuNPs. **a** Effect of concentration of Aspirin, Quercetin and polyethylene glycol 9000 capped and Quercetin functionalized AuNPs on the % inhibition of hemolysis and **b** a standard graph with line of regression showing direct correlation between concentrations of polyethylene glycol 9000 capped and Quercetin functionalized AuNPs and % inhibition of hemolysis

Protein denaturation is well familiar cause for inflammation. Salicylic acids, flufenamic acid and other anti-inflammatory drugs have shown dose dependent ability to inhibit protein denaturation and RBCs membrane stabilization [55]. As a part of our efforts to investigate anti-inflammatory potential, the activity of AuNPs-PG9-QT was studied to inhibit the heat induced hemolysis of RBCs and RBCs membrane stabilization. In this case, the standard drug Asp and control QT at 1600 mg/L showed a maximum of 6.18 and 99.51% inhibitions, respectively while AuNPs-PG9-QT in the range of 200–2000 mg/L was found effective in inhibiting the heat induced hemolysis of RBS to variable extent (Table 2). With increase in the concentration of AuNPs-PG9-QT, the dose dependent inhibition of hemolysis was observed (Fig. 6a). Highest of 91.26% inhibition was achieved at 2,000 mg/L of AuNPs-PG9-QT with IC_50_ value of 287.177 (Fig. 6b). Although the precise mechanism of NPs mediated membrane stabilization is yet to be elucidated, it may be possible that AuNPs-PG9-QT produced this effect by changing the surface area/volume ratio of the cells. This could have caused an expansion of membrane or the shrinkage of the cells and an interaction with membrane proteins [56].

**Table 2:** Effect of concentration of aspirin, quercetin and PG9 capped and quercetin functionalized AuNPs on the % inhibition of hemolysis

Concentration (ppm)		% Inhibition		
		Aspirin	Quercetin	AuNPs-PG9-QT
	200	− 24.70 ± 5.03	7.50 ± 7.64	48.60 ± 8.50
	400	− 17.02 ± 2.98	27.22 ± 6.22	56.33 ± 15.09
	600	− 4.68 ± 2.49	65.05 ± 6.36	59.08 ± 1.65
	800	− 27.60 ± 6.66	89.51 ± 5.49	52.86 ± 3.97
	1000	− 14.81 ± 3.4	93.27 ± 10.89	69.90 ± 3.77
	1200	− 13.58 ± 0.91	95.36 ± 6.53	73.35 ± 8.51
	1400	− 9.26 ± 0.74	97.62 ± 8.19	74.51 ± 21.16
	1600	6.18 ± 1.51	99.51 ± 6.55	87.46 ± 6.83
	1800	3.51 ± 1.3	93.97 ± 8.41	88.52 ± 8.67
	2000	3.48 ± 1.61	96.85 ± 6.67	91.26 ± 6.25
IC50		− 14.195 ± 2.15	94.315 ± 8.71	71.62 ± 6.14

### Anti-angiogenic activity of AuNPs-PG9-CPT

**Fig. 7:**
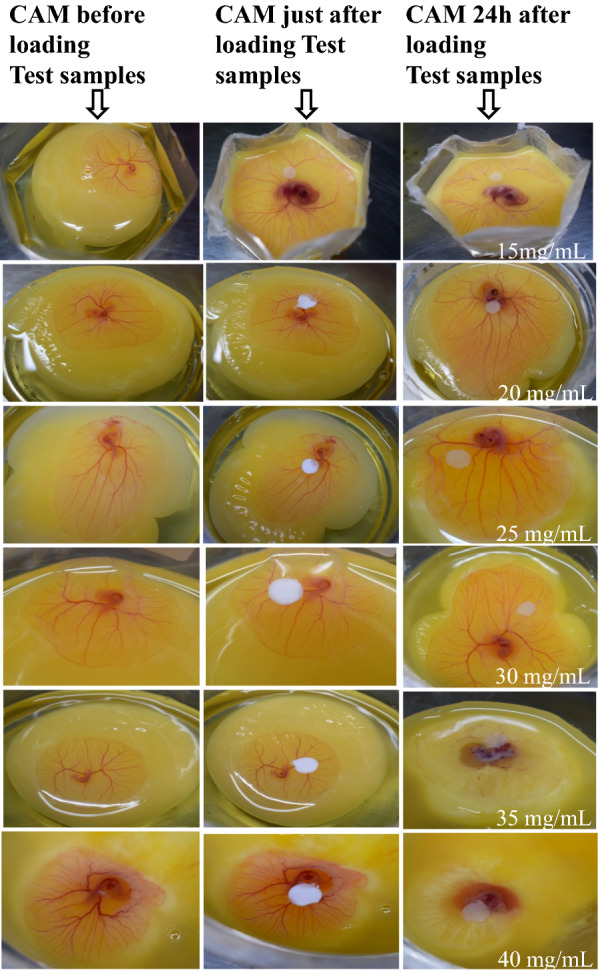
Photographs of CAM showing the effect of polyethylene glycol 9000 capped and camptothecin functionalized AuNPs in the range of 15–40 mg/mL

Angiogenesis is the process of formation of new blood vessels of tissue or organ and anti-angiogenic effects of test samples can be studied by assessing the test sample mediated prevention of growth of new blood vessels [57]. The chicken embryo is well-known animal model and the CAM based assay is widely used [58] as it effectively responds to pro- and anti-angiogenic drugs. This technique is performed either *in-ovo* or shell-less *ex-ovo* [59]. The *ex ovo* CAM assay is a method of assessing the anti-angiogenic potential of various test compound with minimal invasion. In present study, the chicken embryo cultured in a plastic cup (*ex-ovo*) provided a better access to the test site, allowed repeated treatments and multiple test [60]. The suitability of plastic cups based *ex ovo* assay for our study was also evident by the higher efficiency and viability of cultured chick embryos due to easy ventilation through the pores on transparent wraps or parafilm on plastic cups [61]. In addition, three to five days were usually sufficient for this assay. In the present study, untreated hen CAMs (Fig. 7) showed normal vascular architecture with prominently branched-out, well-developed blood vessels indicating no major effect whereas, the treatment of CAMs with AuNPs-PG9-CPT in the range of 15–30 mg/mL resulted in marginal increase in inhibition of the blood vessel branching. The complete inhibition of blood vessel formation was observed in CAM treated with 30 and 40 mg/mL AuNPs-PG9-CPT. After 24 h of loading of 30 and 40 mg/mL AuNPs-PG9-CPT, the blood vessels below the disc were observed to disappear demonstrating excellent anti-angiogenic activity.

## Conclusion

In conclusion, ASCE was successfully employed for optimized, rapid and cost-effective biogenic synthesis of AuNPs. Eight minutes of reaction, incubation at 90 °C, reaction pH of 6, 4 mM of HAuCl_4_ and 4:1 ratio of extract to precursor were the optimum parameter for synthesis of crystalline, spherical AuNPs. The surface capping of AuNPs with PG9 was essential prior to functionalization with QT and CPT. The surface capped AuNPs were best functionalized at 70 °C with QT in 1 h reaction at pH 7 containing 1200 ppm of QT, and AuNPs-PG9:QT ratio of 1:4; whereas, CPT was best functionalized at RT in 1 h reaction with parameters such as pH 12, AuNPs-PG9:CPT ratio of 1:1 and 0.5 mM of CPT. PG9 capped and QT functionalized AuNPs showed significant anti-cancer activity and ability to stabilize the RBCs membrane by inhibiting hemolysis whereas, PG9 capped and CPT functionalized AuNPs inhibited the CAM vascularization demonstrating excellent anti-angiogenic activity. Thus, the present study demonstrates an excellent application of drug functionalized biogenic AuNPs to develop nano-systems which can be used for biomedical applications.

## Materials and methods

### Materials

All the chemicals and reagents used in the present study were of analytical (98% pure) grade and used without any further purification. Mature, fully grown, dry Almond (*Prunus amygdalus* Syn. *Prunus dulcis*) nuts were purchased from the local market of Kolhapur, India. Gold (III) chloride trihydrate (HAuCl_4_.3H_2_O), sodium hydroxide (NaOH), dextrose (C_6_H_12_O_6_), sodium citrate (Na_3_C_6_H_5_O_7_), citric acid (C_6_H_8_O_7_), sodium chloride (NaCl),dimethyl sulfoxide (DMSO, 99%), cell culture media-RPMI1640 supplemented with 10% foetal bovine serum (FBS), 1X Dulbecco’s phosphate buffered saline (DPBS), 0.25% Trypsin–EDTA solution and MTT reagent were purchased from Sigma-Aldrich (Sigma Aldrich, Mumbai, India). Quercetin was procured from Sigma-Aldrich (Sigma Aldrich, Mumbai, India) whereas; polyethylene glycol 6000 and 9000 (PG6 and PG9, respectively) and polyvinyl pyrrolidone K25 (PVP K25) were purchased from SRL (Sisco Research Laboratories Pvt. Ltd. Mumbai, India). Hydrochloric acid (HCL, 35.4%) and absolute ethanol were purchased from Thomas Baker (Thomas Baker Chemicals, Pvt. Ltd. Mumbai, India). All the chemicals and reagents were prepared in sterile double distilled water (SDDW) unless otherwise mentioned.

### Preparation of almond seed coat extract

In this study, leathery, brown seed coat of almond nuts was used to obtain phytochemicals rich extract for biogenic synthesis of AuNPs. Briefly, 200 g of almond nuts were thoroughly washed to remove surface impurities and soaked in SDDW at room temperature (RT) for 24 h. Next day, almonds were observed for swelling and brown skins were hand peeled, washed for 3–4 times with SDDW, and then air dried at RT for 24 h. After complete drying, skins were ground to fine powder using mixer grinder (Bajaj Electricals Limited, Mumbai, India). Thereafter, 8 g of powder was suspended in 150 mL of SDDW, stirred at 60 °C for 1 h in water bath. After incubation, the extract was cooled and filtered through muslin cloth to remove the particulate matter. The filtrate was finally centrifuged at 15,000 rpm for 20 min to obtained clear, light yellow ASCE which was then stored at 4–8 °C until further use.

### Optimization of biogenic synthesis of AuNPs

Initially, to study the ASCE’s potential to synthesize AuNPs, 1 mL of 4 mM HAuCl_4_ precursor solution was mixed with 3 mL of ASCE, incubated at 90 °C for 10 min with continuous stirring, monitored for the change in color from light yellow to dark ruby red as indication of AuNPs synthesis. In addition, AuNPs synthesis was also confirmed by recording the UV–Vis. spectra in the range of 250–800 nm on Biospectrophotometer (Eppendorf, Hamburg, Germany). Further to optimize the ASCE mediated synthesis; effects of variations in different synthesis parameters on AuNPs synthesis were studied. To accomplish this, reaction parameters such as incubation time, temperature, pH, ratio of ASCE:precursor and precursor (HAuCl_4_) concentration were varied in the ranges of 0-10 min, 50–90 °C, 2–10, 1:1–5:1 and 0.25–1.5 mM, respectively. For all these optimization experiments also, reactions were monitored initially bythe change of color and then by recording UV–Vis spectra in the range of 250–800 nm. After optimization, large scale synthesis was carried out using optimized conditions; the colloidal solution of AuNPs was then centrifuged at 25,000 rpm for 15 min to collect AuNPs pellet. The pellet was washed 3–4 times, dried at 60 °C in hot air oven for 12 h, and characterized using different spectroscopy and imaging techniques.

### Characterization of biogenic AuNPs

In this study, optimally synthesized AuNPs were confirmed by recording UV–Vis. spectra whereas the elemental composition was confirmed by energy dispersive X-ray spectroscopy (EDS) using AztecLive EDS analysis software (Oxford Instruments, UK). Further, Fourier transform infrared spectroscopy (FTIR) analyses of dried ASCE and AuNPs were carried out to study the functional groups of different biomolecules of ASCE that could have functioned as reducing and capping agents. To this end, 2 mg each of AuNPs and ASCE were mixed with 200 mg of potassium bromide (KBr) and pellet were prepared by applying pressure of 6–7 tons to obtain pellets of 1–1.5 mm thickness. FTIR spectra were then recorded in transmittance mode over the range of 400–4000 cm^−1^ on FTIR spectrometer (Bruker alpha Shimadzu, Japan). The X-ray diffraction (XRD) pattern analysis was employed to study the crystalline nature of AuNPs by recording XRD pattern on Bruker’s AXS analytical instruments (Bruker Pvt. Ltd., Germany). In addition, size, morphologyand selected area electron diffraction pattern (SAED) of AuNPs were imaged using high resolution transmission electron microscope (HR-TEM) operating at an acceleration voltage of 200 kV (Jeol Ltd., Tokyo, Japan). The average size of AuNPs was estimated by dynamic light scattering (DLS) and zeta potential (surface charge) measurement was done by PALS zeta potential analyser var. 5.76 (Brookhaven Instrument Corp. Holtsville, New York, USA).

### Surface capping and functionalization of AuNPs with quercetin and camptothecin

In order to facilitate and enhance the functionalization of AuNPs with QT and CPT, initially three different polymers namely PG6, PG9 and PVP K25 were screened and evaluated for their potential utility in surface capping prior to functionalization. Briefly, freshly prepared 1 mL of 0.1 M of aqueous solutions of PG6, PG9 and PVP K25 were added separately to the 1 mL of neat AuNPs. The solutions were stirred at RT for 24 h to allow the complete exchange of AuNPs surface molecules with capping agents. To confirm the pegylation and capping of AuNPs with PVP K25, the preparations were analyzed by recording UV–Vis. spectra in the range of 250–800 nm. During capping reaction, red shift of the SPR band resulting from increased refractive index surrounding the AuNPs due to the modification of capping agent was monitored [44]. The preparations showing maximum absorbance with redshift in the SPR were selected as successfully capped and used for functionalization with QT and CPT.

To functionalize the capped AuNPs (CAuNPs) with QT (CAuNPs-QT), an initial reaction was carried out by mixing CAuNPs with QT (1000 ppm) in 1:3 ratio, pH 6 and stirred at 50 °C for 2 h. In order to optimize the functionalization reaction parameters and study how the variations in different parameters such as incubation time, pH, temperature, concentration of QT and CAuNPs:QT ratio influence the functionalization, they were varied in the ranges of 0–120 min, 4–7, 30–70 °C, 1:1–1:4 and 400–1600 ppm, respectively. In case of functionalization of CAuNPs with CPT (CAuNPs-CPT), initially 0.5 mM CPT in 5% DMSO was mixed with CAuNPs in 1:1 ratio, pH 7 and stirred at RT for 1 h. Later, the functionalization conditions were optimized by studying the effects of variations in reaction time, concentration of CPT, ratio and pH etc. in the ranges of 1–6 h, 0.2–0.5 mM, ratio 1:1 and pH 2–12 respectively. To confirm the functionalization, the reactions were initially analyzed by recording UV–Vis. spectra throughout the experiments. After optimization, large scale functionalization were done using optimized parameters, CAuNPs-QT and CAuNPs-CPT were then centrifuged at 20,000 rpm for 15 min and pellets were washed with SDDW to remove unbound QT, CPT and organic impurities. Further, CAuNPs-QT and CAuNPs-CPT were dried 40 °C, characterized using EDS and FTIR spectroscopy and stored at RT until further use.

### Anti-cancer activity of quercetin functionalized AuNPs

The anti-cancer activity of AuNPs-PG9-QT was tested against Human Breast Cancer Cell line MCF-7 using MTT assay. Briefly, MCF-7 cells cultured in T-25 flasks were trypsinized and aspirated into a 5 mL centrifuge tube. Cell pellet was obtained by centrifugation at 300 × g, cell count was adjusted using DMEM HG medium such that 200 μL of suspension contained approximately 10,000 cells. To each well of the 96 well micro-titre plate, 200 μL of the cell suspension was added and the plate was incubated at 37 °C and 5% CO_2_ atmosphere for 24 h. After 24 h, the spent medium was aspirated, 200 μL of test samples such as QT, AuNPs and AuNPs-PG9-QT to the final concentrations in the range of 25–800 μg/mL were added to the respective wells. The standard drug Cisplatin was also included in the assay. The plate was then incubated at 37 °C and 5% CO_2_ atmosphere for 24 h. Thereafter, the plate was removed from the incubator, the drug containing media was aspirated, 200 μL of medium containing 10% MTT reagent was added to each well to get a final concentration of 0.5 mg/mL and then the plate was incubated at 37 °C, 5% CO_2_ atmosphere for 3 h. Next, the culture medium was removed completely without disturbing the crystals formed, 100 μL of solubilization solution (DMSO) was added and the plate was gently shaken in a gyratory shaker to solubilize the formed formazan. The absorbances were measured using a microplate reader at a wavelength of 570 nm and also at 630 nm. The percentage growth inhibition was calculated after subtracting the background and the blank, and concentration of test sample needed to inhibit cell growth by 50% (IC_50_) was estimated from the dose–response curve for the cell line. The percentage (%) of cell viability was calculated using following formula:

$$\% {\text{cell viability}} = \frac{{{\text{Absorbance of sample}}}}{{{\text{Absorbance of control}}}} \times 100 \%$$

### Anti-inflammatory activity of quercetin functionalized PG9 capped AuNPs

The anti-inflammatory activity of AuNPs-PG9-QT was assessed by employing the membrane stabilization test based on inhibition of heat induced hemolysis of RBCs. To accomplish this, fresh human blood was collected at Health Centre Hospital of Shivaji University, Kolhapur by following the standard medical practices and mixed with equal volume of sterilized Alsever’s solution (2% dextrose, 0.8% sodium citrate, 0.05% citric acid and 0.42% sodium chloride). The mixture was then centrifuged at 3000 rpm for 10 min, the packed cells were washed thrice with saline (0.85%, pH 7.2), and the obtained blood volume was measured and reconstituted as 10% (v/v) suspension with saline. For the test of anti-inflammatory activity, the heat induced hemolysis reactions consisted of 1 mL of 10% RBCs suspension and 1 mL of AuNPs-PG9-QT to the final concentrations in the range of 200–2000 mg/L. Saline solution was added instead of test sample to the blank test tube while Aspirin (Asp) and QT were used as standard and control drugs, respectively. All the reactions were incubated in a water bath at 56 °C for 30 min., cooled under running tap water and centrifuged at 2500 rpm for 5 min. The absorbance of supernatant was recorded at 560 nm. The experiments were performed in triplicates for all test samples. The % inhibition of hemolysis was calculated by using the formula given below:

$$\% {\text{inhibition}} = \frac{{{\text{Absorbance of control}} - {\text{Absorbance of sample}}}}{{{\text{Absorbance of control}}}} \times 100\%$$

### Anti-angiogenic activity of camptothecin functionalized AuNPs

For the test of CAM based anti-angiogenic activity of AuNPs-PG9-CPT, zero-day old eggs were obtained from the Egg hatchery center, Kolhapur, India. All the handling and experimental steps were performed in laminar air flow (LAF) hood and all tools were cleaned and surface sterilized with 70% ethanol. Prior to the CAM assay, the outer surfaces of the eggs were cleaned with SDDW, placed vertically into egg incubator and incubated at 37 °C and 70% humidity for 72 h. After 72 h, eggs were kept horizontally for one hour without rotation to bring the CAM on upper side of the egg and facilitate the transfer of an intact embryo to the plastic cups for *ex-ovo* culture. Thereafter, the eggs were removed from the incubator, transferred to LAF hood, cracked open gently using metal bar and the contents were carefully transferred to the surface sterilized plastic cups. Further, the plastic cups were covered with parafilm, the *ex-ovo* cultures were returned to the egg incubator with minimal disturbances and dead embryos were removed to minimize infection on the CAM and increase their survival rates. Next day, the numbers of blood vessels of the embryos were counted prior to the application of the filter paper discs loaded with test samples. For efficient observation of the effects of test samples on the angiogenesis, discs were placed directly using a sterile forceps over the blood vessels on the growing CAM where two major blood vessels bifurcated. To this end, the circular discs were loaded with AuNPs-PG9-CPT in the range of 15–40 mg/mL along with standard drug CPT (0.5 mM). Subsequently, the *ex-ovo* cultures were returned to the egg incubator, and the final evaluations were carried out on day 5. The number of blood vessels was counted at the site of sample application, photographed and the comparison was done before and after treatment.

## Supplementary Information

**Additional file 1**: **Figure S1**. UV-Vis spectra surface capping reactions of AuNPs with different capping agents. **Figure S2**. UV-Vis absorption spectra showing effect of different (a) AuNPs-PG9-QT synthesis, (b) Incubation times, (c) pH, (d) ratio of AuNPs-PG9:QT, (e) incubation temperature and (f) concentration of QT on AuNPs-PG9-QT synthesis. **Figure S3**. UV-Vis absorption spectra showing effect of different physico-chemical parameters (a) concentration of CPT, (b) incubation times, (c) pH on AuNPs-PG9-CPT synthesis. **Table S1**. Fourier transform-infrared spectroscopy based analysis of ASCE and AuNPs for the study of vibrational stretchings and corresponding functional groups. **Table S2**. Fourier transform-infrared spectroscopy based analysis of QT and AuNPs-PG9-QT for the study of vibrational stretchings and corresponding functional groups (DOCX 1802 KB)

## Data Availability

All data generated or analysed during this study are included in this published article and its supplementary information files.

## Abbreviations

ASCE: Almond seed coat extract
SDDW: Sterile Double distilled water
NPs: Nanoparticles
MNPs: Metal nanoparticles
NMs: Nanomaterials
RT: Room temperature
AuNPs: Gold nanoparticles
SPR: Surface plasmon resonance
Au: Elemental Gold
PG9: Polyethylene glycol 9000
PG6: Polyethylene glycol 6000
PVP K25: Polyvinyl pyrrolidoneK25
AuNPs-PG6: PG6 capped Gold nanoparticles
AuNPs-PG9: PG9 capped Gold nanoparticles
AuNPs-PVP K25: PVP K25 capped Gold nanoparticles
CAuNPs: Capped AuNPs
QT: Quercetin
AuNPs-PG9-QT: Quercetin functionalized PG9 capped Gold nanoparticles
CAuNPs-QT: QT functionalize capped AuNPs
CPT: Camptothecin
CAuNPs-CPT: CPT functionalize capped AuNPs
AuNPs-PG9-CPT: Camptothecin functionalized PEG 9000 capped Gold nanoparticles
MTT: 3-(4,5-Dimethylthiahiazol-2-yl)-2,5-diphenyl tetrazolium
Asp: Aspirin
RBCs: Red blood cells
PBS: Phosphate Buffered Saline
CAM: Chorioallantoic membrane assay
