# Supplementary material for: Optimally biosynthesized, PEGylated gold nanoparticles functionalized with quercetin and camptothecin enhance potential anti-inflammatory, anti-cancer and anti-angiogenic activities
Source: J Nanobiotechnology. 2021 Mar 25;19:84. doi: 10.1186/s12951-021-00836-1 (PMC7992809; doi:10.1186/s12951-021-00836-1)
Supplement: Supplementary file 1 — Additional file 1: Figure S1. UV-Vis spectra surface capping reactions of AuNPs with different capping agents. Figure S2. UV-Vis absorption spectra showing effect of different (a) AuNPs-PG9-QT synthesis, (b) Incubation times, (c) pH, (d) ratio of AuNPs-PG9:QT, (e) incubation temperature and (f) concentration of QT on AuNPs-PG9-QT synthesis. Figure S3. UV-Vis absorption spectra showing effect of different physico-chemical parameters (a) concentration of CPT, (b) incubation times, (c) pH on AuNPs-PG9-CPT synthesis. Table S1. Fourier transform-infrared spectroscopy based analysis of ASCE and AuNPs for the study of vibrational stretchings and corresponding functional groups. Table S2. Fourier transform-infrared spectroscopy based analysis of QT and AuNPs-PG9-QT for the study of vibrational stretchings and corresponding functional groups (DOCX 1802 KB) [file 12951_2021_836_MOESM1_ESM.docx]

**Supplementary material**

**Figure S1** UV-Vis spectra surface capping reactions of AuNPs with different capping agents

**Figure S2.** UV-Vis absorption spectra showing effect of different (a) AuNPs-PG9-QT synthesis, (b) Incubation times, (c) pH, (d) ratio of AuNPs-PG9:QT, (e) incubation temperature and (f) concentration of QT on AuNPs-PG9-QT synthesis

**Figure S3.** UV-Vis absorption spectra showing effect of different physico-chemical parameters (a) concentration of CPT, (b) incubation times, (c) pH on AuNPs-PG9-CPT synthesis.

**Table S1** Fourier transform-infrared spectroscopy based analysis of ASCE and AuNPs for the study of vibrational stretchings and corresponding functional groups.

| **Frequency cm^-1^** | **Functional group with type of vibrations** |
| --- | --- |
| 3403.94 and  3219.01 | O-H, N-H stretching in amine group and Hydroxyl functional group bonded to the aromatic ring |
| 2922.07 and 2853.75 | C-H stretching vibrations |
| 2355.98 and 2091.00 | C=C and C-C stretching |
| 2357.81 | C=Cstretch bonded |
| 1737.89 | stretching vibrations of carbonyl (C=O) and aldehyde groups |
| 1641.42 and 1635.55 | N–H bending of primary amine |
| 1455.10 | C–H bending vibrations of the aromatic tertiary amine group |
| 1385.18 | Methyl groups (alkanes) of bioactive molecules |
| 1109.35 | –C–O– stretching vibrations of carboxylic |
| 1023.95 | C–N stretching vibration of aliphatic amines or to alcohols/phenols |
| 916.02 and 860.07 | O–H bend of carboxylic acid and C-X stretching in alkyl halides |
| 665.90 | –CH=CH bending vibrations of ethylene systems |
| 638.00 | C-C bending |

**Table S2** Fourier transform-infrared spectroscopy based analysis of QT and AuNPs-PG9-QT for the study of vibrational stretchings and corresponding functional groups.

| **Frequency cm^-1^** | **Functional group with type of vibrations** |
| --- | --- |
| 3305.77 | H-bonded OH groups in phenolic and |
| 2977.46 and 2924.71 | C-H stretching vibrations |
| 1636.00 and 1642.72 | N–H bending of primary amine |
| 1511.79 | C–C stretch in-ring due to aromatic group |
| 1459.42 | C–H bending vibrations of the aromatic group |
| 1345.96 | O–H bend in phenol |
| 1249.95 | C–O stretch characteristic of phenol |
| 1084.11 | C–O stretch attributed to secondary alcohol |
| 1040.11 | C–N stretching vibration of aliphatic amines of phenols |
| 953.19 | C–H bend due to aromatic hydrocarbon |
| 883.36 | N–H bend due to primary and tertiary amine |
| 665.16 | C–H bending vibration of the aromatic group |
| 647.70 | Phenolic ring bending of quercetin |
